# Treatment of Pigmented Basal Cell Carcinoma with 3 mm Surgical Margin in Asians

**DOI:** 10.1155/2016/7682917

**Published:** 2016-08-29

**Authors:** Shang-Hung Lin, Yu-Wen Cheng, Yi-Chien Yang, Ji-Chen Ho, Chih-Hung Lee

**Affiliations:** Department of Dermatology, Kaohsiung Chang Gung Memorial Hospital and Chang Gung University College of Medicine, Kaohsiung, Taiwan

## Abstract

*Background.* In Asians, most basal cell carcinomas (BCCs) are pigmented with clear borders. The consensus of 4 mm surgical margin for BCC largely depends on studies in nonpigmented BCCs in Caucasians. However, little is known about recurrences of pigmented BCCs with a narrower surgical margin. We aimed to investigate 5-year recurrence of BCCs, either pigmented or nonpigmented, in Taiwanese with 3 mm surgical margin.* Materials and Methods.* 143 patients with BCC (M/F = 66/77, average 64 years) were confirmed pathologically from 2002 to 2013. Based on the pathological margin (>1 mm, ≤1 mm, and involved), patients were categorized into the complete excision group (*n* = 77), histology with close proximity group (*n* = 43), and unclear surgical margin group (*n* = 23).* Results.* Among 143 cases, 105 were pigmented. With standard 3 mm excision, there were 7 recurrences, with 6 of them from nonpigmented BCC group. Logistic regression showed that pigmentation was associated with lower recurrence. Interestingly, 5-year recurrence of completely excised and histology with close proximity BCC (0/77 versus 1/43) was not different statistically.* Conclusions.* A 3 mm surgical margin is adequate for pigmented BCC. A “wait and see” approach rather than further wide excision is appropriate for BCC with <1 mm free margin.

## 1. Introduction 

Basal cell carcinoma (BCC) is the most common type of skin cancer [1]. Clinically, it may present as a pigmented nodule with a rolling border and telangiectasia on sun-exposed areas, especially the face and neck. Management of BCC includes Mohs micrographic surgery, standard surgical excision, destruction by various modalities, and topical chemotherapy [2]. Because local recurrence rather than distant metastasis is the main clinical concern for excised BCC, the gold standard for BCC treatment is complete resection with clear margins.

Most BCCs in Asians are pigmented (52.4%–90%), and the gross margin is thus usually discernible [3–7]. Although Mohs micrographic surgery has been used for the purpose of tissue sparing for facial BCC, standard excision with a 3 mm margin is frequently performed in our practice, because of the obvious border of pigmented BCCs.

The National Comprehensive Cancer Network (NCCN) recommends a relatively wide margin (≥4 mm) for BCC [2]. Kimyai-Asadi et al. reported that narrow margins (1–3 mm) were inadequate for the excision of small, well demarcated, primary nodular BCCs on the face [8]. Thomas et al. suggested 4 mm margin for BCCs and 3 mm margin for well demarcated BCCs [9]. The meta-analysis from Gulleth et al. suggested that 3 mm surgical margin can be safely used for nonmorpheaform basal cell carcinoma [10]. However, most patients in previous studies were White, a population that seldom develops pigmented BCC.

In the treatment of pigmented BCC, Aoyagi and Nouri reported using an average margin of 3.89 mm in Mohs micrographic surgery [11]. Ito et al. reported that a margin of 2 to 3 mm was adequate for well-defined, primary pigmented, and nonmorpheaform BCC [7]. However, long-term outcomes for BCC excision with narrow margins have not been reported. The present study used a 5-year follow-up design to determine whether a 3 mm surgical margin was appropriate for excision of pigmented and nonpigmented BCC with the recurrence as the outcome measurement. In addition, because of an urgent need to decide clinically whether further excision should be performed when the histologic examination shows close proximity, we investigated recurrence rates for completely excised BCC, histology with close proximity, and unclear surgical margins in excised BCC.

## 2. Materials and Methods

**Figure 1:**
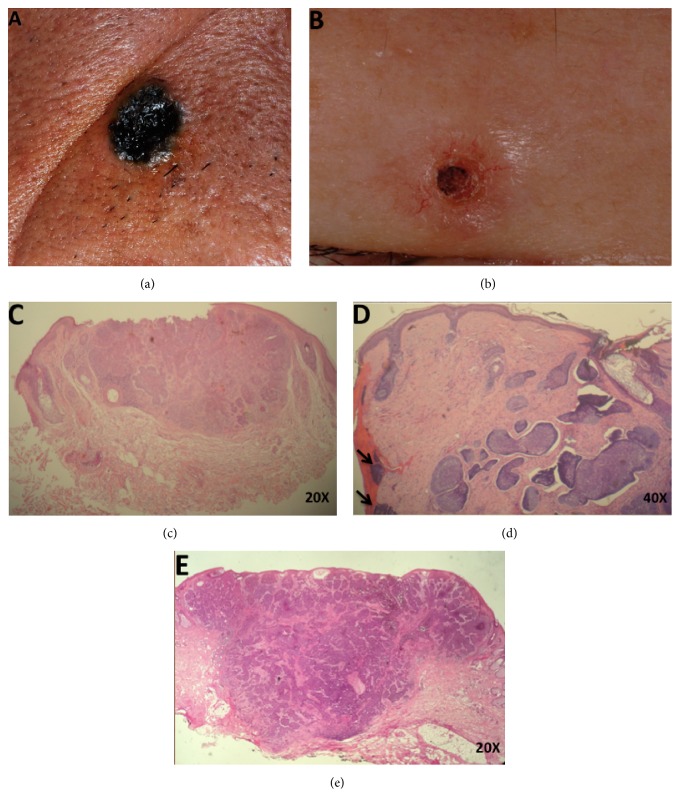
(a) Pigmented BCC; (b) nonpigmented BCC; (c) completely excised BCC; (d) BCC with unclear surgical margins (arrows); (e) histology with close proximity BCC.

This study was approved by the Institutional Review Board of Chang Gung Memorial Hospital (104-7368B). We retrospectively reviewed the records of 143 patients who received a histological diagnosis of BCC during the period from 2002 to 2013. All patients had undergone surgery with a standard 3 mm excision at least 1 year before. Postoperatively, all specimens got completely serial section to confirm both bilateral and deep margins in histology. Pigmented and nonpigmented BCCs were differentiated on the basis of the presence or absence of gross pigmentation (Figures 1(a) and 1(b)). On the basis of pathology reports using 1 mm as the cut point, the complete excision group was defined cases with a margin greater than 1 mm (Figure 1(c)). The unclear surgical margin group (Figure 1(d)) was defined as residual tumor on excision margin. The close proximity group (Figure 1(e)) was defined as the lateral or deep excision margins within 1 mm. Patients with morpheaform BCC were excluded. If the pathological findings showed unclearly excised BCC, we explained the pathological report, the odds of recurrence, and the possibility of reoperation to the patients. After full discussion, these patients preferred the observation and may receive operation if the tumor recurred. Clinical data, including gender, age, time of diagnosis, tumor size, tumor pigmentation, tumor location, clinical outcome, and BCC recurrence, were ascertained from medical records.

Surgical outcomes were compared between patients with pigmented and nonpigmented BCC. Age, sex, tumor size, follow-up duration, location, and recurrence were analyzed by the chi-square or *t*-test, to analyze associations between groups. Outcomes were also compared for patients with unclearly, histology with close proximity, and completely excised BCC. Age, sex, tumor site, size, and pigmentation and duration of follow-up were analyzed by chi-square or *t*-test, to investigate associations with recurrence among the three groups. A *p* value less than 0.05 was considered to indicate statistical significance in all analyses. Risk factors for BCC recurrence were determined by univariate analysis followed by multiple logistic regression adjusted for age and sex.

## 3. Results

### 3.1. Recurrence Was Higher in Nonpigmented BCC Than That in Pigmented BCC

A total of 143 patients with histologically confirmed BCC were identified (males/females: 66/77; mean age: 64 years). There were 105 pigmented BCCs and 38 nonpigmented BCCs. The nonpigmented BCC group had a slightly higher proportion of women (65.8%) as compared with the pigmented BCC group (49.5%) (*p* = 0.06). Average age was similar in the pigmented and nonpigmented BCC groups (63.9 and 65.3 years, resp.). Tumor size was similar (1.43 and 1.26 cm, resp.), as was duration of follow-up (5.4 and 4.7 years, resp.). As compared with other facial regions, the nose was the most frequent site for nonpigmented BCC. One patient with pigmented BCC (micronodular type) (1/105, 1%) and 6 with nonpigmented BCC group (4 micronodular types and 2 nodular types) (6/38, 15.8%) developed recurrence. The recurrence rate was higher for nonpigmented BCC than for pigmented BCC (*p* = 0.001) (Table 1). The higher recurrence rate for nonpigmented BCC group was comparable to that reported in a study that found that the recommended narrow margin was inadequate for excision of nonpigmented BCC in Whites.

### 3.2. Demographic Characteristics

To determine whether further excision or a “wait and see” approach was indicated for histology with close proximity BCC, we investigated recurrence rates for cases of completely excised BCC, histology with close proximity, and unclear surgical margins in excised BCC (Table 2). Seventy-seven patients had completely excised BCC (males/females: 38/39; average age: 63 years): 61 pigmented BCCs and 16 nonpigmented BCCs. Twenty-three patients had unclearly excised BCC (males/females: 8/15; average age: 68 years): 10 pigmented BCCs and 23 nonpigmented BCCs. Forty-three patients had histology with close proximity BCC (males/females: 20/23; average age: 65 years): 20 pigmented BCCs and 23 nonpigmented BCCs. The nose was the most common site for patients with completely excised BCC and BCC with unclear surgical margins, while the cheek was the most common site for histology with close proximity BCC. Mean tumor diameter was similar among the groups: 1.64, 1.62, and 1.34 cm for completely excised BCC, histology with close proximity BCC, and BCC with unclear surgical margins, respectively. Mean duration of follow-up was 5.3, 4.5, and 5.5 years, respectively. Patient age, sex, tumor size, and duration of follow-up did not differ significantly among the groups. Of note, recurrence developed in no patients with completely excised BCC (0/77, 0%) and only in 1 patient with histology with close proximity BCC (1/43, 2.3%), as compared with 6 patients with BCC with unclear surgical margins (6/23, 26%). The recurrence rate for patients with BCC with unclear surgical margins was significantly higher as compared with the other 2 groups; however, the difference between patients with completely excised and histology with close proximity BCC (0% versus 2.3%) was not significant. The percentage of nonpigmented BCCs was higher for patients with unclear surgical margins than for those with completely excised and histology with close proximity BCC. There was no statistical difference in relation to pigmentation between patients with completely excised and histology with close proximity BCC. Nonpigmented BCCs may have unclear tumor borders and thus a tendency to have unclear surgical margins. In addition, BCCs above the ear were more likely to have unclear surgical margins.

### 3.3. Lower Risk of Recurrence for Pigmented BCC

We used univariate and multiple logistic regression to determine whether pigmentation was associated with the risk of recurrence. Univariate analysis showed that unclear surgical margins and pigmentation were risk factors for BCC recurrence. More importantly, multiple logistic regression analysis showed that pigmented BCC had a much lower risk for recurrence, after adjustment for age and sex (95% confidence interval: 0.006–0.475; *p* = 0.008; Table 3). The data indicated that pigmentation is a major important prognostic factor in Asians with pigmented BCC after excision.

## 4. Discussion

Our results show that Taiwanese BCC patients were older and tended to have pigmented BCC and to be male. Recurrence was more frequent for nonpigmented BCC than for pigmented BCC (15.8% versus 1%, *p* = 0.001). The recurrence rate was higher for patients with unclear surgical margins than for those with completely excised and histology with close proximity BCC. However, the recurrence rate was similar for the latter two groups. Multiple logistic regression analysis showed that pigmented BCC was associated with lower risk for recurrence, after adjustment for age and sex. These results suggest that a 3 mm surgical margin is appropriate and advisable for pigmented BCC. In addition, the recurrence rates for histology with close proximity BCC and completely excised BCC were similar. We also found that BCC near the ear was associated with unclear surgical margins.

The optimal surgical margin for BCC is a matter of controversy. Wolf and Zitelli reported that a margin of 4 mm was adequate for 95% of nonmorpheaform BCCs smaller than 2 cm in diameter, when treated by means of standard excision [12]. The National Comprehensive Cancer Network (NCCN) recommends a relatively wide margin (≥4 mm) [2]. Kimyai-Asadi et al. reported that BCC excision with 1, 2, and 3 mm margins was associated with positive margins in 16%, 24%, and 13% of tumors, respectively. They concluded that narrow margins (1–3 mm) are inadequate for excision of small, well demarcated, primary nodular BCCs of the face [8]. Thomas et al. suggested 4 mm for basal cell carcinoma and 3 mm margin for well demarcated BCCs [9]. On the other hand, the meta-analysis from Gulleth et al. suggested that 3 mm surgical margin can be safely used for nonmorpheaform basal cell carcinoma with 2 cm or smaller [10]. However, these recommendations were based on studies of nonpigmented BCCs. The optimal surgical margin for pigmented BCC remains uncertain. Yeh et al. reported that prevalence rates for pigmented BCC were lower for BCC with a depth greater than 3.3 mm, as compared with a depth less than 3.3 mm, and suggested that excision of BCCs without pigmentation should be deeper for Taiwanese patients [6]. Goh et al. reported that pigmented BCC was more frequently excised with adequate margins than were nonpigmented tumors with comparable histologic subtypes [13]. Aoyagi and Nouri reported that the total mean surgical margin was smaller for pigmented BCCs than for nonpigmented BCCs (3.89 mm versus 5.85 mm; *p* < 0.05) [11]. In a Japanese study, Ito et al. reported a 95.3% cure rate for a 2 mm margin and a 100% cure rate for a 3 mm margin [7]. However, these studies lack data on long-term outcomes.

In Taiwan, most BCCs are pigmented, and 73.4% (105/143) of the present BCCs were pigmented. The usual margin for BCC excision in our department is 3 mm, and the recurrence rate for pigmented BCCs was 1% after an average follow-up period of 5.4 years. This encouraging result may be due to the clearer tumor borders of pigmented BCC. In addition, the conspicuousness of pigmented skin lesions may lead patients to seek care earlier than those with nonpigmented BCCs. A 3 mm free margin results in good clinical outcomes and preserves most normal tissue and is thus optimal and adequate for excision of pigmented BCC.

Controversy remains regarding management of histology with close proximity BCCs. Longhi et al. reported that, among 40 patients with tumors “in close proximity” (<1 mm), only 1 (2.5%) developed recurrence after 6 years of follow-up. The authors concluded that a clear margin and histology with proximity were associated with a low chance of relapse, while unclear margins significantly increased the risk of recurrence and justified immediate reexcision [14].

The recurrence rate for histology with close proximity BCCs was 2.3% after an average of 4.5 years of follow-up in our study. The recurrence rate did not differ significantly for patients with histology with proximity and completely excised BCC. Pigmented BCC was associated with lower risk of recurrence in multiple logistic regression, which may be related to the higher percentage of pigmented BCCs in our study. Our results indicate that a “wait and see” approach is advisable for histology with close proximity BCCs.

The canthus, nasolabial fold, and periorbital and postauricular areas are high-risk anatomic sites for BCC and should be treated with Mohs micrographic surgery. Mulvaney et al. reported that ear BCCs appear to exhibit greater subclinical extension than do BCCs not involving the ear, head, or neck [15]. Therefore, the ear should also be considered a high-risk location for BCCs. Our results showed that the percentage of BCCs over the ear was higher in patients with unclear surgical margins than in the other groups. Therefore, we suggest more aggressive excision and Mohs micrographic surgery for BCCs located near the ear.

This study may have limitations. First, although the follow-up duration was 62 months, a longer follow-up period may be even better for evaluating BCC recurrence, which can occur as late as a decade later. Second, this is a study limited to patients from one clinic in Taiwan and may not be generalizable to the entire world.

## 5. Conclusions

The present results suggest that a 3 mm margin is adequate for excision of pigmented BCC. Nonpigmented BCC had a higher risk for recurrence and thus warrants careful follow-up. The recurrence rate for histology with close proximity BCC was not significantly higher than that for completely excised BCC. Therefore, a “wait and see” approach rather than further wide excision is advisable for histology with close proximity BCC.

## Figures and Tables

**Table 1:** Demographics of patients with pigmented and nonpigmented BCCs.

	Pigmented BCC	Nonpigmented BCC	*p* value
Number	105	38	

Males/females	53/52	13/25	0.06

Average age (years)	63.9	65.3	0.92

Average tumor diameter (cm)	1.43	1.26	0.244

Follow-up (years)	5.4	4.7	0.289

Location			
Scalp	2	2	0.287
Forehead	8	1	0.255
Ear	0	2	0.069
Nose^*∗*^	27	17	0.026
Nasolabial fold	6	4	0.256
Periorbital areas	18	3	0.131
Cheeks	27	6	0.154
Lips	4	0	0.286
Trunk	10	2	0.334
Limbs	3	1	0.713

Recurrence (%)^*∗*^	1/105 (1%)	6/38 (15.8%)	0.001

^*∗*^ *p* value < 0.05.

**Table 2:** Demographics of patients with completely excised BCC, histology with close proximity BCC, and BCC with unclear surgical margins.

	Completely excised BCC	Histology with close proximity BCC	BCC with unclear surgical margins
Number	77	43	23

Males/females	38/39	20/23	8/15

Average age (years)	63	65	68

Pigmented/nonpigmented^*∗*^	61/16	34/9	10/13

Location			
Scalp	3	0	1
Forehead	7	1	1
Ear^*∗*^	0	0	2
Nose	22	13	9
Nasolabial fold	5	4	1
Periorbital areas	10	7	4
Cheeks	17	13	3
Lips	3	1	0
Trunk	6	4	2
Limbs	4	0	0

Tumor diameter (cm)	1.64	1.62	1.34

Follow-up (years)	5.53	4.48	5.5

Recurrence (%)^*∗*^	0/77 (0%)	1/43 (2.3%)	6/23 (26.0%)

Recurrence time (years)	NA	7.6	3.23

^*∗*^ *p* value < 0.05.

**Table:** (a) Univariate analysis

*p* value	Recurrence
Age	0.911
Sex	0.342
Unclear surgical margins^*∗*^	0.000
Tumor diameter	0.918
Location	0.213
Pigmentation^*∗*^	0.000

^*∗*^ *p* value < 0.05.

**Table:** (b) Multiple logistic regression

	*p* value (recurrence)	95% confidence interval
Age	0.957	0.934–1.067
Sex	0.639	0.114–3.801
Pigmentation^*∗*^	0.008	0.006–0.475

^*∗*^ *p* value < 0.05.
